# Editorial: Current status of honey bee genetic and breeding programs: progress and perspectives

**DOI:** 10.3389/finsc.2024.1365665

**Published:** 2024-01-29

**Authors:** María Alejandra Palacio, Ernesto Guzman-Novoa, Alejandra Carla Scannapieco, Agostina Giacobino, Fanny Mondet

**Affiliations:** ^1^ Estacion Experimental Agropecuaria (EEA) Balcarce, Instituto Nacional de Tecnología Agropecuaria (INTA), Instituto de Innovación para la Producción Agropecuaria y Desarrollo Sostenible (IPADS), Consejo Nacional de Investigaciones Científicas y Técnicas (CONICET), Facultad de Ciencias Agrarias – Universidad National de Mar del Plata (FCA-UNMdP), Avignon, France; ^2^ School of Environmental Sciences, University of Guelph, Guelph, ON, Canada; ^3^ Instituto de Genética, Instituto Nacional de Tecnología Agropecuaria (INTA), Instituto de Agrobiotecnología y Biología Molecular (IABIMO) Consejo Nacional de Investigaciones Científicas y Técnicas (CONICET), Hurlingham, Buenos Aires, Argentina; ^4^ Estacion Experimental Agropecuaria (EEA) Rafaela, Instituto Nacional de Tecnología Agropecuaria (INTA), Instituto de Investigacion de la Cadena Láctea (IDICAL) -Consejo Nacional de Investigaciones Científicas y Técnicas (CONICET), Santa Fé, Argentina; ^5^ French National Institute for Agriculture, Food and Environment (INRAE), Abeilles et Environnement, Avignon, France

**Keywords:** *Apis mellifera* (honeybee), selection, Varroa resistance, africanized honey bees, genetic markers, honey bee characterization

Honey bees (*Apis mellifera* L.) contribute to food production and sustain biodiversity in managed and natural ecosystems through the pollination of a variety of plant species, which helps maintain a dynamic equilibrium among animals and plants. Additionally, honey bees have been bred by humans because of the value of their products. Populations of these insects face numerous challenges that affect their fitness and survival as the intensification of agriculture, climate change, and some diseases and parasites, including the mite *Varroa destructor*. The economic value of honey bees has attracted scientific and technical interest and their unusual genetic and behavioral attributes have increased the interest of geneticists and breeders. Nowadays, advances in molecular characterization of honey bee traits have greatly expanded our knowledge of this species and bring a unique opportunity for updating the criteria of selection and preservation of honey bee genetic resources. Resistance has been one of the main selection criteria applied worldwide, and gentleness had special attention in the Americas where Africanized honey bee (AHB) populations are present. Characterization of genetic materials is a key point in selection programs and new tools have been developed for this purpose in recent years.

This Research Topic includes 6 published articles that focused on the genetic characterization, identification, and breeding of honey bee populations, as well as on tools, traits, and genetic markers, for the selection of *Varroa* resistence and defensive behavior of honey bees. Litvinoff et al., applied a morphometric approach and haplotype analyses on drones and workers in an area of natural hybridization in Argentina, sampling bees not only from colonies but also from Drone Congregation Areas (DCA) to monitor hybridization between AHB and European honey bees (EHB).

Avalos and Bilodeu used a microfluidic platform and machine learning analyses to develop a new genotyping assay that capitalizes on markers used for genetic identification of Russian honey bees, and that could be used for breeding decisions.

Tarpy et al., used a meta-analysis approach to compare pedigree relationships and to quantify the overall genetic diversity of a feral AHB population, an experimental inbred population, and three commercially managed populations. Results obtained were used to analyze the population genetic diversity of managed honey bees in the USA and to suggest priorities to be considered in breeding programs.

Bianchi et al., developed a protocol and an index for the selection of AHB stocks based on defensive behavior, and characterized contrasting colonies for this trait using Next Generation Sequence (NGS) technologies. They identified SNPs related to genes previously associated with defensive behavior and discussed the influence of polyandry and paternal lineages on this behavior in AHB, and the use of dRADseq to assist in the selection and evaluation of honey bee stocks showing low defensive behavior for commercial purposes.

Gabel et al., using an extensive dataset of Buckfast and Carniolan stocks of honey bees in Germany, investigated the heritabilities and genetic correlations of suppressed mite reproduction (SMR) and recapping as resistance traits against *Varroa destructor*. They discuss the relationship between individual predictivity and response to selection for different traits that affect the reproductive ability of the parasite, as well as difficulties in quantifying these parameters. They suggest that *Varroa* resistance is difficult to quantify using these parameters for selective breeding on a larger scale.

Morfin et al., studied the genetic mechanisms underlying self-grooming behavior in high and low *Varroa* population growth honey bee genotypes. They conducted gene expression and viral quantification analyses and identified differentially expressed genes (DEGs) associated with the two honey bee genotypes. Results suggest a possible involvement of odorant binding protein genes in the perception of irritants (eg., *Varroa* mites), which would trigger rapid self-grooming in honey bees.

In summary, this Research Topic delved into the multifaceted aspects that are or could be applied in honey bee breeding programs. The outcomes of the studies evidenced numerous tools that could be used to characterize honey bee populations and/or to assist honey bee selective breeding, with *Varroa* resistance of honey bee colonies being one of the main drivers of research for scientists and breeders. The availability of honey bee genotypes that are resistant to *V. destructor* may not only contribute to honey bees’ vitality and decrease colony losses, but also reduce the use of synthetic acaricides by incorporating breeding as part of an Integrated Pest Management (IPM) strategy.

Despite these significant advancements, just a few of them are applied in commercial breeding programs and only in specific cases. Nevertheless, the use of this knowledge in the production of honey bee stocks will increase benefits for the beekeeping industry worldwide.

We extend our gratitude to all authors for their insightful contributions, as well as to the reviewers and the editorial team.

## Author contributions

MAP: Writing – original draft. ACS: Writing – review & editing. EG-N: Writing – review & editing. AG: Writing – review & editing. FM: Writing – review & editing.

